# Simulations of Protein Adsorption on Nanostructured Surfaces

**DOI:** 10.1038/s41598-019-40920-z

**Published:** 2019-03-18

**Authors:** Berardo M. Manzi, Marco Werner, Elena P. Ivanova, Russell J. Crawford, Vladimir A. Baulin

**Affiliations:** 1 Universitat Rovira i Virgili, Departament d’Enginyeria Quimica, Tarragona, 43007 Spain; 2 Leibniz-Institut für Polymerforschung Dresden e.V., Institut Theorie der Polymere, Dresden, 01069 Germany; 3 RMIT University, School of Science, Melbourne, VIC 3001 Australia

## Abstract

Recent technological advances have allowed the development of a new generation of nanostructured materials, such as those displaying both mechano-bactericidal activity and substrata that favor the growth of mammalian cells. Nanomaterials that come into contact with biological media such as blood first interact with proteins, hence understanding the process of adsorption of proteins onto these surfaces is highly important. The Random Sequential Adsorption (RSA) model for protein adsorption on flat surfaces was modified to account for nanostructured surfaces. Phenomena related to the nanofeature geometry have been revealed during the modelling process; e.g., convex geometries can lead to lower steric hindrance between particles, and hence higher degrees of surface coverage per unit area. These properties become more pronounced when a decrease in the size mismatch between the proteins and the surface nanostructures occurs. This model has been used to analyse the adsorption of human serum albumin (HSA) on a nano-structured black silicon (bSi) surface. This allowed the Blocking Function (the rate of adsorption) to be evaluated. The probability of the protein to adsorb as a function of the occupancy was also calculated.

## Introduction

Recent progress in the miniaturization of electronics at the nanoscale^1^ and the development of experimental techniques allowing the visualization of nanostructures below the diffraction limit^2,3^, has allowed the design of certain nanostructures on substrata, the properties of which can now be precisely controlled. Materials possessing nanostructured surfaces have also shown great potential in the manufacture of medical implants, because they exhibit not only mechano-bactericidal properties, but also have been shown to facilitate an increased level of contact between a substrate and the surrounding tissues^4–7^. Nanostructured surfaces occur naturally on lotus leaves, insect wings, shark skin, gecko feet, fish scales and spider silk, and in each case the surface provides the substrate with a natural barrier to bacterial adhesion^8^. Mimicking the behavior of such surfaces to engineer artificial surfaces that have similar properties would allow the manufacture of bacteria-free medical implants, and have applicability to protein chip technology and other biomedical fields.

The growing demand for implant biomaterials and biomedical devices with antibacterial nanostructured surfaces requires a more accurate quantitative description and prediction of their interaction with the biological environment. Proteins, in particular, are some of the first compounds to come into contact with the surface of biomaterials. Protein adorption has the potential to reduce the antimicrobial properties of nanostructured surfaces, and because this phenomenon is not well understood, the interaction requires an in-depth investigation^9^.

Protein adsorption on surfaces has been the subject of long-standing debate, principally because of the wide range of adsorption mechanisms adopted by diverse protein structures. Proteins differ in size and shape, have variable conformational states in solution, and exhibit different kinetics of adsorption^10,11^. The nature of protein interactions with surfaces ranges from weak Van-der-Waals interactions to strong electrostatic attraction; proteins that are fast diffusing, but interact to a lesser extent with the substratum, can readily be replaced by slow-diffusing and strongly interacting proteins via the so-called ‘Vroman effect’^12,13^. Apart from the complex adsorption kinetics that can exist, cooperative adsorption between proteins^14^ may lead to the development of even more complex kinetics under physiological conditions such as those found in blood plasma, which contains more than 1000 proteins^15^. This plethora of interaction phenomena makes it difficult to construct a generalized theoretical framework that is capable of describing protein adsorption at the molecular level.

Despite this complexity, there are general models available that can succesfully describe protein adsorption at a coarse-grained level. One of the most popular models used to describe protein adsorption is the Langmuir adsorption model. This model was initially developed for adsorption of gases onto porous materials^16^. Classical Langmuir adsorption is limited to a single species adsorbing onto a flat surface that contains a finite number of adsorption sites. The model makes several assumptions that may not be realistic in real systems. These are: (i) all adsorption sites are equivalent; (ii) only one molecule can adsorb onto a given site at any time; (iii) no interaction is present between the adsorbing molecules; and (iv) the process of adsorption is reversible. Despite these assumptions potentially limiting the validity of the model, its simplicity allows the model to be applied to a broad range of systems that do not meet these assumptions, including, in most cases, protein adsorption. Experimental data can often appear to be compatible with a Langmuir adsorption isotherm, with “effective” equilibrium constants able to be obtained from the modelling process, although the underlying mechanism of adsorption is not necessarily reversible. Hence, the resulting values obtained from the model of surface saturation and the adsorption constant can be misleading as a consequence of an erroneous interpretation, as previously demonstrated by Latour^17^, and emphasized again in the present work.

Random Sequential Adsorption (RSA)^18^ is a well-established comprehensive theoretical framework for describing irreversible particle adsorption. It is also known as the “*Car parking problem*” (in 1 dimension). This model approaches adsorption as a stochastic process, in which particles are sequentially placed onto a surface upon which other particles are already present. An attempted adsorption event will fail if the condition of adsorption is not met. In a simple 2D hard disk model adsorption, a disk is successfully adsorbed if it does not overlap with any of the previously adsorbed particles (steric repulsion). Adsorption conditions have also been formulated for non-spherical particles, mixtures and different adsorption acceptance criteria^19^.

Common features extracted from the RSA approach are the saturation occupancy, *i.e*. the surface concentration *θ*_∞_ for *t* → ∞, and the finite time necessary to reach the surface concentration, up to a certain precision (*e.g*. 0.99*θ*_∞)_, as a function of the number of attempts. The dependence of these quantities on adsorption rates and the diffusion constant requires a generalization of the RSA model, and introducing kinetic rate or diffusion equations^20^. This type of approach allows the geometrical aspects of the classical RSA simulation to be linked to the physical quantities describing the adsorption dynamics or, for example, the bulk concentration required to achieve a certain RSA configuration on the surface.

The aim of the current study is to take the classical models for protein adsorption on flat surfaces and modifying them so that they are applicable to the 3D scale of the nanostructured surfaces. To this end, we developed an extension of the RSA approach^18^, applying it to the different types of nanostructures (such as pillars, spikes and curved cones) that can be found on natural templates of the nanostructured surfaces, such as those of the dragonfly and cicada wings and/or their synthetic analogues such as black silicon (bSi)^7,21^. Since human serum albumin (HSA) is one of the most abundant proteins in blood plasma and the first adsorbate in the Vroman process^12,13^, we used HSA as the model protein in the development of the 3D RSA model. The model was then applied to a nanostructured bSi substrate surface.

Developing the model allowed us to extract several pieces of information, such as the amount of protein required to saturate the surface as a function of the convexities, together with the typical time scales of adsorption for different initial bulk protein concentrations. These quantities could readily be related to experimental measurements and imaging information. Furthermore, the linear relationship that exists between the saturation occupancy and the curvature derived at the end of each section 0.2, provides important hints regarding the relationship between the typical nanofeature sizes and the final configuration of proteins on the surface, which is of great relevance in the design of such surfaces.

The work is structured as follows: first, the model, in particular RSA, is discussed, highlighting some important geometrical considerations that are used in subsequent sections. Secondly, the results obtained in the simulations performed on both model surfaces and on real bSi surface were described. We then estimated the time scales involved in this type of processes and finally some conclusive comments are made.

## Methods

### Generalized RSA model for the adsorption of protein

The kinetics of protein adsorption for a given bulk concentration *n* of proteins is commonly expressed through the fraction of covered area *θ* as a function of time *t*. It is usually assumed that the adsorption of proteins does not appreciably affect the bulk protein concentration. This serves as a model control parameter, along with the rates of adsorption and desorption, *k*_*a*_ and *k*_*d*_, respectively. The kinetics of adsorption are described by the following equation^22^:

1 $$\frac{d\theta }{dt}={k}_{a}nB(\theta )-{k}_{d}\theta $$

Here, *B*(*θ*) is the *blocking function*, also known as the *surface exclusion effect function*. This is defined as the probability to adsorb a protein to the surface for a given occupancy *θ*. For an empty surface, e.g. at the beginning of adsorption, *θ* = 0 and the blocking function *B*(*θ*) = 1. As the surface coverage increases during the adsorption process, *B*(*θ*) decreases as less substrate surface area becomes available for adsorption, and thus the probability of finding an empty adsorption site decreases. Equation (1) describes the transition towards a dynamic equilibrium, which depends on the concentration and, as we show in the following sections, on the geometrical features of the underlying surface, which determine the shape of the blocking function. Note that the adsorption step described by *B*(*θ*) is only a part of the dynamics at the surface. Within this picture, the surface acts as a “sink”, and the bulk solution provides an infinite supply of proteins. A more in-depth analysis would require coupling the RSA approach to the diffusion equation, which would lead to proper consideration of timescales.

In general, the shape of *B*(*θ*) is not trivial and requires further assumptions to be made regarding the protein shape and adsorption mechanism. The simplest model used to describe protein adsorption kinetics is the Langmuir adsorption model. It assumes a blocking function of the form *B*(*θ*) = *θ*_∞_ − *θ*, which allows an analytical solution for (1) to be determined:

2 $$\theta =\frac{{K}_{eq}n}{1+{K}_{eq}n}{\theta }_{\infty }$$

where *K*_*eq*_ = *k*_*a*_/*k*_*d*_. Here *θ*_∞_ is called the *jamming limit*, *i.e*. the maximum surface coverage *θ*. No further particles can adsorb to the surface beyond this threshold. The assumptions of reversibility of the Langmuir adsorption process, however, are generally incompatible with the experimentally determined protein adsorption concentrations, the mechanism of which is almost or completely irreversible. Therefore, a more general form for *B*(*θ*) is necessary. (For a more thorough comparison between the RSA model and the Langmuir adsorption model, please refer to the Supplementary Materials).

### Geometrical considerations

The following discussion emphasizes the role of geometry in the process of protein adsorption. To illustrate this concept, we consider adsorption onto both a flat and spherical substrate, where it becomes clear how the surface occupancy *θ* is dependent upon the surface curvature.

**Figure 1:**
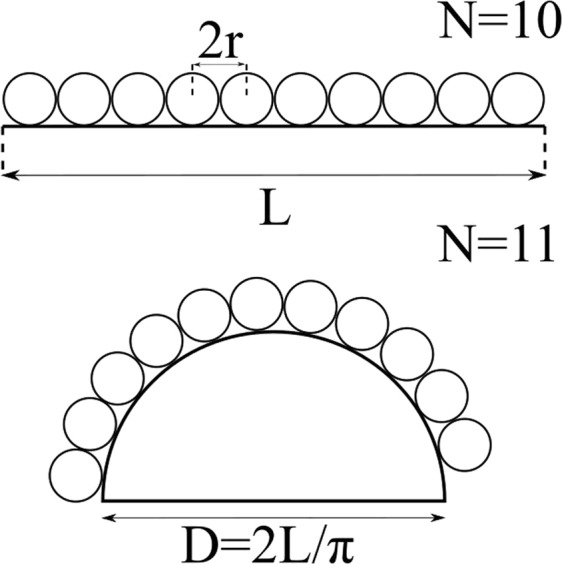
Comparison of sphere occupancy in one dimension on a flat surface and on a spherical shell. The given length *L* of the segment in the upper case (flat substrate) corresponds to 2*L* = *πD* in the lower case (curved substrate), where *D* is the diameter of the hemisphere. In both figures, the small spheres have identical diameters, but in the second case the occupancy *θ* is larger.

Figure 1 shows two type of geometries: a flat 1D “surface” (i.e. a line segment), and a semicircle, both of length *L*. The different occupancy of spherical proteins on top of both surfaces is evident from the image, since in the first case, 10 spheres of diameter *r* = *L*/20 fit ono the surface, whereas in the second case, it is possible to arrange 11 spheres on the surface without completely filling the semicircle. Despite this type of description becoming less obvious in higher dimensions, the rationale generally holds, and therefore we expect the final occupancy *θ*_∞_ to be strongly dependent on these effects.

### Random sequential adsorption onto nanostructured surfaces

A characteristic feature of the RSA model is the inherent assumption that the adsorption process is governed by surface exclusion, *B*(θ). In general, an analytical solution to the RSA adsorption is not feasible and therefore *Monte Carlo* (MC) simulations of the adsorption process are utilized. MC simulations provide direct access to the blocking function *B*(*θ*) and *jamming limit θ*_∞_. In our MC simulations, the blocking function *B*(*θ*) at a given coverage *θ* in the RSA model is defined as the ratio between the number of potentially successful attempts, *N*_*succ*_, and the total amount of attempts, *N*_*att*_^23^.

3 $$B(\theta )=\frac{{N}_{succ}}{{N}_{att}}$$

For hard disks on a flat surface, the jamming limit yields^19,24^ *θ*_∞_ = 0.547 ± 0.002, while the blocking function can be expressed to a third order as^22^ $B(\theta )=1-4\theta +\frac{6\sqrt{3}}{\pi }{\theta }^{2}+(\frac{40}{\pi \sqrt{3}}-\frac{176}{3{\pi }^{2}}){\theta }^{3}+{\mathscr{O}}({\theta }^{4}).$ Our approach is in line with the method of Schaaf and Talbot^22^, which assumes that only steric repulsion is present between the spheres. Each system is simulated *N* times and for each configuration in a given simulation, the number of attempts required to adsorb the *i*-th particle is computed. Numerically, the calculation is performed with the use of a MC method, which records the number of successful attempts required to attach the protein onto a free site. Each simulation is repeated 1000 times. As a result, the blocking function *B*(*θ*) is computed as an average of the number of attempts for a given surface coverage *θ*. For further details, refer to the Supplementary Materials.

In order to illustrate our findings with a practical example, we focussed on the adsorption of HSA, the most abundant protein in blood plasma^25^ and very common in experimental studies^9^. Although crystallographic data depicts HSA as a heart-shaped protein^26^, in solution the excluded volume surrounding the protein screens its non-spherical shape. In fact, there is good agreement between Quartz Crystal Microbalance (QCM) experiments and models of spherical proteins^27^ and, thus, HSA is generally treated as a sphere in theoretical modelling^28^. Hence, our coarse grained approach models albumin proteins as sterically repelling spherical particles of diameter *D* = 7 *nm*, which represents the typical size of albumin proteins^29^.

To gain an insight into the effect of nanostructure curvature at the surfaces, we can illustrate the effect with model shapes described by only a few parameters, then extend this knowledge in the modelling of adsorption onto real surfaces possessing irregular shapes. Thus, in our model, the nanostructured surfaces are presented by an array of equidistant periodic Gaussian structures:

4 $${f}_{i}(x,y)=(\begin{array}{ll}H\,\exp (-\frac{{(x-{x}_{i})}^{2}+{(y-{y}_{i})}^{2}}{2v}) & {\rm{if}}{(x-{x}_{i})}^{2}+{(y-{y}_{i})}^{2}\le \frac{{d}_{p}^{2}}{4}\\ 0 & {\rm{otherwise}}\end{array}$$

for −*d*_*p*_/2 < *x* < *d*_*p*_/2 and −*d*_*p*_/2 < *y* < *d*_*p*_/2. Here, *H* denotes the height of the structures. With this, *C* = (*x*_*i*_, *y*_*i*_) is the center of the *i*-th peak, *d*_*p*_ is the peak-to-peak distance, and the variance of the Gaussian $v={(\frac{W}{2})}^{2}\frac{1}{2\,\mathrm{log}(2)}$, where *W* is the width at half height. Such a description is rather general, but can be used to provide an insight into the differences in adsorption that take place on different classes of surfaces, since it limits the number of parameters used to describe the surface topology. In other words, we utilize the generic expression 4 for curved surface to emphasize the importance of surface topology in protein adsorption, and we include more detailed parameters in order to provide concrete examples of existing nanostructures^7,21^.

In the following discussion, we consider four distinct classes of “nanostructures”: (i) Flat surfaces, (ii) Gaussian pillars, (iii) Gaussian spikes and (iv) holes. The Gaussian pillars being considered here represent a fair approximation of the nanopillars found on cicada wings (with *H* = 200 *nm* and *W* = 80 *nm*)^21^, whereas the Gaussian spikes correspond dimensionally to those found on the surface of Black Silicon (with *H* = 500 *nm* and *W* = 60 *nm*)^7^.

**Figure 2:**
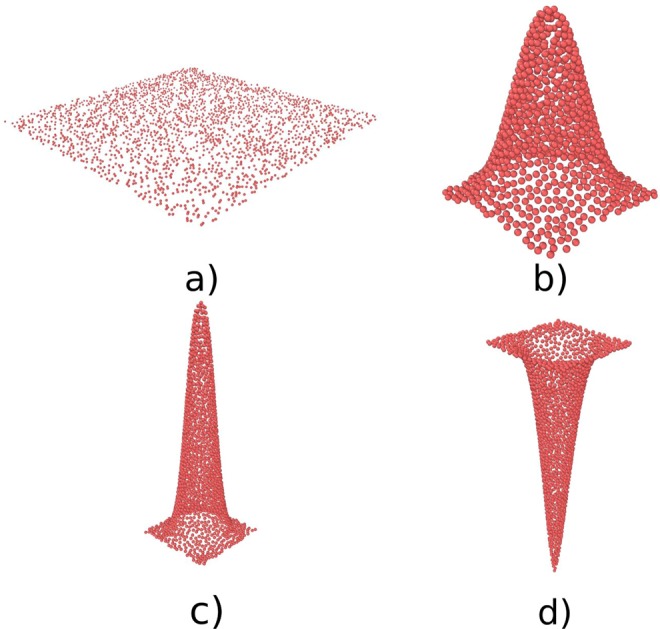
Intermediate (**a**) and final (**b**–**d**) configuration for RSA simulations of four different surfaces: (**a**) Flat surface, (**b**) Gaussian pillar with *W* = 80 nm and *H* = 200 nm (*Psaltoda claripennis* model), (**c**) Gaussian spike with *W* = 60 nm and *H* = 500 nm (bSi model) and (**d**) Gaussian “hole” with *W* = 60 nm and *H* = −550 nm. This snapshots have been obtained using OVITO^31^.

In order to determine the relationship between the extent of adsorption and the nature of the geometrical features on the surfaces, we performed 7 sets of simulations with different widths and heights of Gaussian spikes and pillars. The simulations using the Gaussian hole was performed to determine the adsorption behavior of proteins onto a “concave” analogue of a Gaussian spike, with an opposite behavior. Snapshots of the final configuration of the RSA simulations on some of these surfaces are shown in Fig. 2.

**Figure 3:**
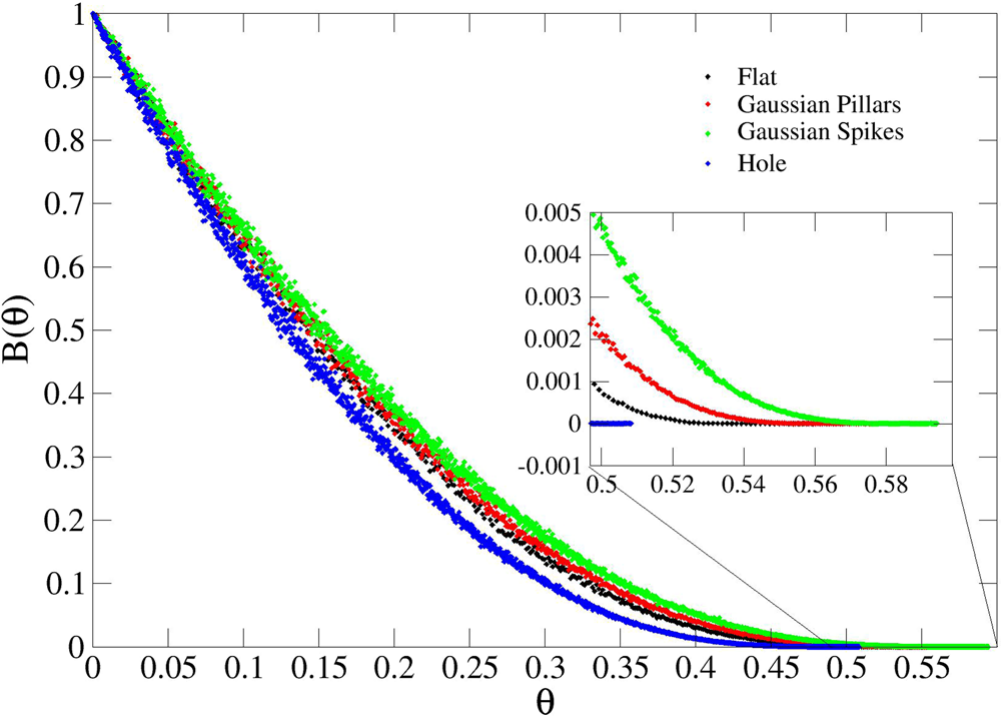
Blocking function *B*(*θ*) for: flat surface (black), Gaussian pillars (red), Gaussian spikes (green) and Gaussian hole (blue) defined by Equation (4). The inset highlights the ending points corresponding to *θ*_∞_.

A comparison of the blocking functions for four different geometries is shown in Fig. 3. Here, the right tail of *B*(*θ*) is shifted towards greater values as the height of the spike increases, confirming that the topologies that consist of bulk material surrounded by empty space favor adsorption compared to that onto empty space surrounded by bulk material (*e.g*. holes). The *jamming limit*, computed as the average final value over all simulations for each configuration, follows the same trend, as reported in Table 1. The jamming limit for the flat surface obtained by J. Tablot and P. Schaaf^22^ is reported for reference, showing the consistency of our results.

**Table 1:** Jamming limit *θ*_∞_ for different geometries: flat surface, Gaussian pillars, Gaussian spikes and Gaussian holes. The values are obtained as the average of the coverage in the final state for each set of simulation. The error bars are estimated as standard deviation of each set. The values in the last column are the relative increase of occupancy by adsorbed proteins with respect to the flat surface, Δ*θ*_∞_ = (*θ*_*∞,s*_ − *θ*_*∞,f*_)/*θ*_*∞,f*_.

	H (nm)	W (nm)	*A*_*s*_/*A*_*f*_	*θ*_∞_	Δ*θ*_∞_(%)
Flat	—	—	1	0.545 ± 0.007	—
Flat (Schaaf *et al*.^22^)				0.547 ± 0.002	
Gaussian pillars	150	80	1.7	0.558 ± 0.005	2.4
	200	60	1.9	0.568 ± 0.005	4.2
	200	70	2.0	0.565 ± 0.005	3.7
	200	80	2.1	0.562 ± 0.004	3.1
	200	90	2.2	0.561 ± 0.004	2.9
	200	100	2.2	0.560 ± 0.004	2.8
	250	80	2.5	0.566 ± 0.004	3.9
Gaussian spikes	450	60	3.3	0.581 ± 0.004	6.6
	500	40	2.8	0.597 ± 0.004	9.5
	500	50	3.2	0.588 ± 0.003	7.9
	500	60	3.6	0.582 ± 0.003	6.8
	500	70	3.9	0.577 ± 0.003	5.9
	500	80	4.2	0.574 ± 0.003	5.3
	550	60	3.8	0.583 ± 0.003	7.0
Gaussian Hole	−550	60	3.8	0.499 ± 0.003	−8.4

**Figure 4:**
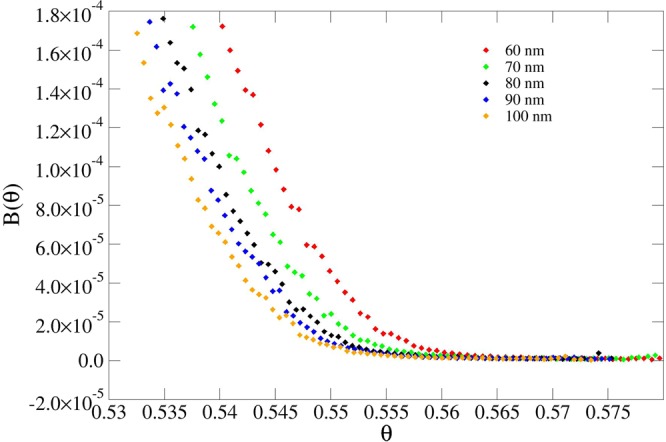
Blocking function of pillars using different widths at fixed height (200 nm).

**Figure 5:**
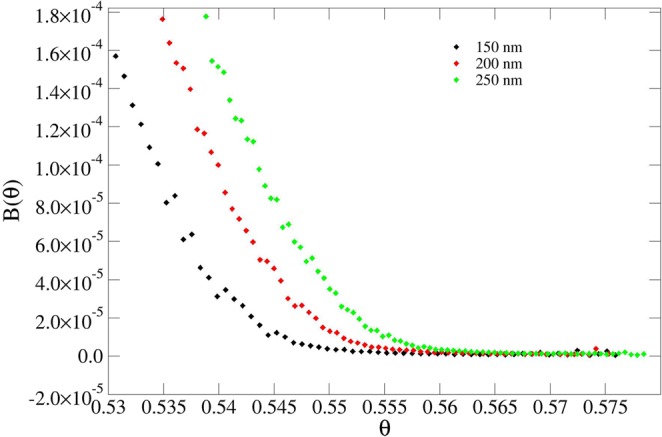
Blocking function of pillars using different heights at fixed width (80 nm).

**Figure 6:**
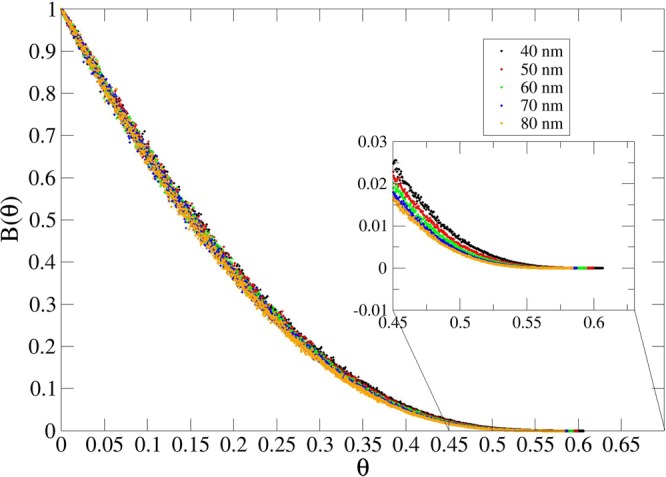
Blocking function of spikes using different widths at fixed height (500 nm). The isolated points are due to fluctuations in the statistical sampling.

**Figure 7:**
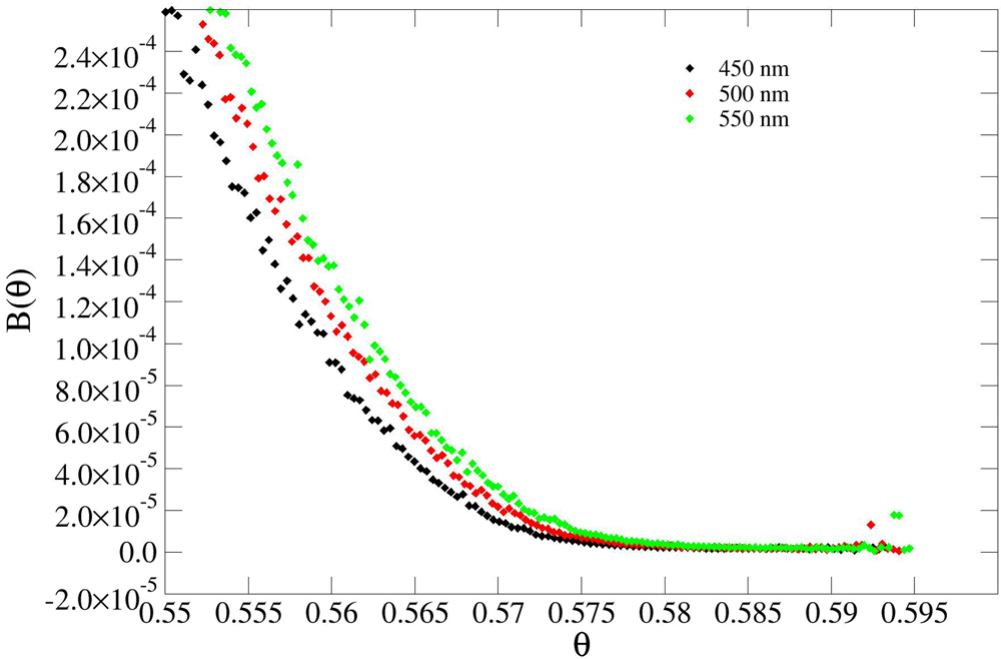
Blocking function of spikes using different heights at fixed width (60 nm). The isolated points are due to fluctuations in the statistical sampling.

The results presented in Table 1 demonstrate that the jamming limit *θ*_∞_ is very sensitive to the width *W* (or, equivalently, to the curvature) of the pillars, at any given height, while it is less sensitive to the changes in the height of the pillars. This is expected due to the larger size mismatch between the pillar height and protein particle dimension. Figures 4–7 show this phenomenon in the behavior of the blocking function *B*(*θ*).

Evaluating the blocking function allows the equation (1) to be solved using a proper fit of the plots obtained so far. Following Talbot and Schaaf^22^, we use a third order polynomial to fit *B*(*θ*) for each of the simulation sets. The resulting parameters are presented in Table S1 of the Supplementary Materials.

Expansion of the blocking function *B*(*θ*) to the third order, as explained by P. Schaaf and J. Talbot^22^, allows information to be obtained regarding the surface exclusion effects of *k*-tuplets of spheres. The first order term is the most relevant at low coverage, with no or few pairs contributing to the magnitude of *B*(*θ*). Therefore from Fig. 3, for *θ* → 0, we observe a faster decrease in surface availability, for flat surfaces or holes, confirming the greater occurrances of occupancy of a single sphere. The second order term takes into account the overlap of pair exclusion areas, which becomes relevant at higher density, while the third order defines the overlap of area for three particles. The latter is, in our approximation, the leading term for *θ* → ∞, as it is responsible of the deviation of the four curves from each other at high coverage. This effect arises due to the higher packing levels achieved for spikes and pillars, which is, in turn, caused by the lower occupancy of spheres on the surfaces containing higher degrees of curvature.

Following the discussion about curvature effects in Section 0.1, it is important to re-emphasize the importance of geometrical aspects in the problem of protein adsorption. In fact, occupancy *θ* is a surface area independent quantity, thereby, if the blocking function and, consequently, the isotherms were merely dependent on this quantity, the jamming limit *θ*_∞_ would not change. Moreover, from inspection of the data presented in Table 1, the variations of *θ*_∞_ as a function of the width *W* are quite significant, especially in the case of Gaussian spikes we see a variation of about 4% when going from *W* = 40 *nm* to *W* = 80 *nm*. Conversely, a greater mismatch between the protein size and the pillar width affects the jamming limit to a lower degree, as can be observed by comparison of *θ*_∞_ for a flat surface with that applicable to Gaussian pillars, where *W* = 100 *nm*. Consequently, the increase of the number of adsorbed proteins on nanopatterned surfaces is larger than that which would have occurred had only the surface area increased, as shown by Δ*θ*_∞_ in the last column of Table 1. The relative ratio between the number of adsorbed proteins on a flat nanostructered surface is given by:

5 $$\frac{{N}_{s}}{{N}_{f}}=\frac{{A}_{s}{\theta }_{{\rm{\infty }},s}}{{A}_{f}{\theta }_{{\rm{\infty }},f}}$$

**Figure 8:**
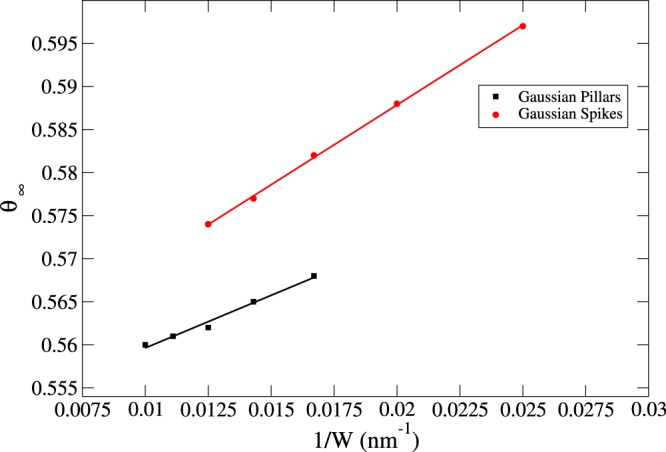
*θ*_∞_ as a function of 1/*W* (i.e. the curvature) for both Gaussian pillars (black) and Gaussian spikes (red). The lines represent a linear fit, demonstrating a linear increase with increase of curvature.

Here, Eq. (5) takes into account the ratio between surface occupancies. The equation shows the importance of geometrical effects in addition to that associated with an increase in surface area (Δ*θ*_∞_ would be 0 (Table 1) if it were only dependent on area increase). Alternatively, Fig. 8 represents the plot of *θ*_∞_ as a function of 1/*W*, i.e. the curvature of the cross-sectional circle at half-height. From the fitted lines, it is reasonable to assume a linear dependency of *θ*_∞_ on the curvature.

**Figure 9:**
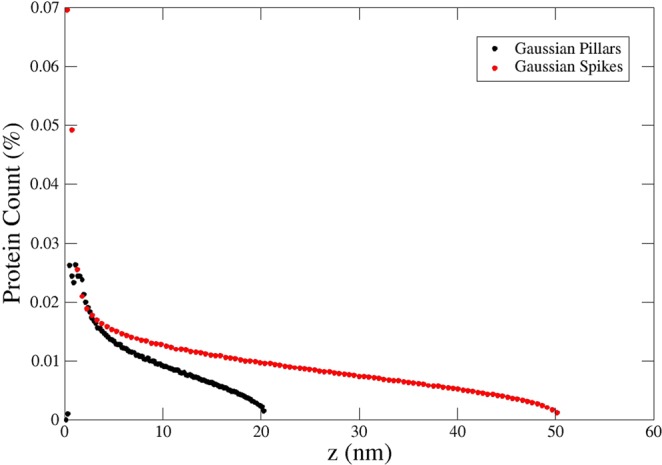
Vertical distribution of proteins for Gaussian pillars with *W* = 80 nm and *H* = 200 nm, and for Gaussian spikes with *W* = 60 nm and *H* = 500 nm. The number of proteins adsorbing on the bottom of the pillars is clearly much greater than that on the top of the pillars, a consequence of lower surface availability. The discontinuity in the left-hand side figure is a simple consequence of the definition of the shape, Eq. 4.

Further insight can be obtained by studying the protein distribution as a function of the vertical coordinate *z*, Fig. 9. The data presented illustrates that the amount of adsorbed proteins is much greater on the bottom of the pillars/spikes than on their top, with this quantity decreasing gradually with *z*. Since the various positions of the proteins have been uniformly generated on the surface (see Supplementary Materials), this trend is a consequence of the available surface area decreasing with *z*. it is noteworthy that this behaviour is in line with previously reported experiments^9^, although no assumption of a “two-step adsorption process” was necessary. The major adsorption on the bottom of the surface is a natural consequence of the greater availability of substrate surface. Moreover, the observations made by D. H. K. Nguyen *et al*.^9^ regarding fibronectin adsorption suggest that for larger proteins, conformational changes indeed play a significant role in the adsorption process (which would necessitate additional analysis with respect to the present work), but confirms that conformational change effects are negligible for small globular proteins such as HSA.

The discussion thus far has focused on model surfaces that possess surface characteristics with dimensions inspired by real surfaces. In the next section, we will apply our findings to a real surface, the details of which have been obtained using Atomic Force Microscopy (AFM) and illustrate the results of our simulations on such surfaces. Some timescales will also be estimated in order to allow comparison with experimental data.

## HSA adsorption onto black silicon surfaces

### RSA on bSi surfaces

**Figure 10:**
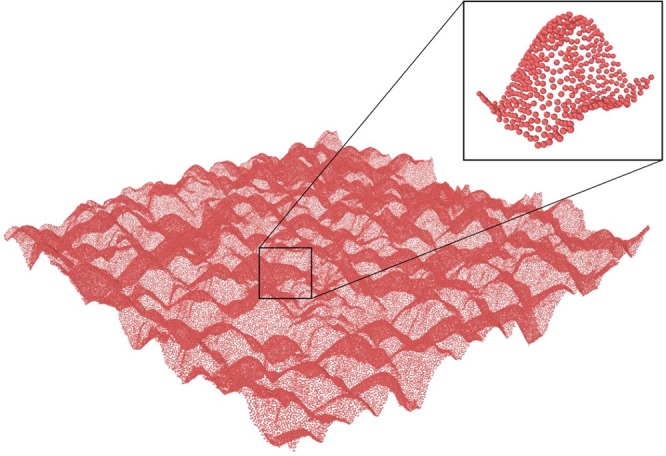
Saturation limit of RSA simulation of albumin adsorption onto a bSi surface represented by an AFM scan.

The approach used for RSA simulations of HSA adsorption onto real bSi surfaces is analogous to the approach outlined in the previous section (and the Supplementary Materials). The surface consists of a 2.5 *μm* × 2.5 *μm* bSi scan, as shown in Fig. 10. AFM scans were performed in tapping mode on an Innova scanning probe microscope (Veeco, Bruker, Billerica, MA), in air at ambient conditions, using a silicon cantilever (Cont20A, Veeco Probes) with a spring constant of 0.9 N/m and a resonance frequency of ~20 kHz. The sample surfaces were first scanned with a 10 *μ*m × 10 *μ*m field of view to ensure even surface coverage and to avoid damaged areas (data not shown), before selecting 1 *μ*m × 1 *μ*m areas for scanning and analysis as described in H. K. Webb *et al*.^30^. Finally, for computational efficiency, the aforementioned 2.5 *μm* × 2.5 *μm* surface was extracted for the sake of subsequent computer simulations.

**Figure 11:**
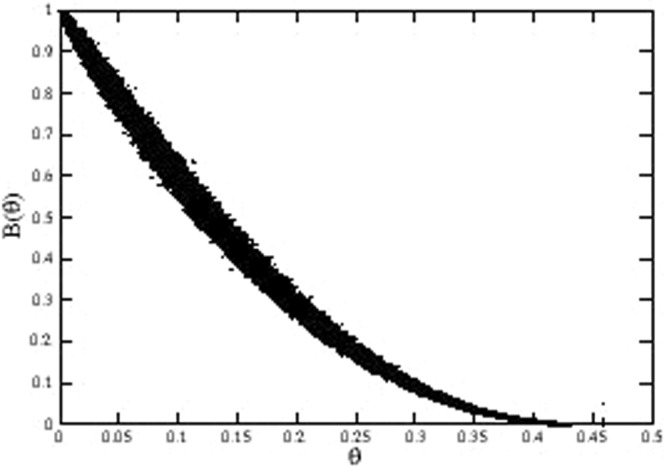
Blocking function of albumin adsorption on the AFM scan of the real bSi surface.

The corresponding Blocking function *B*(*θ*) is reported in Fig. 11, which shows that the final occupancy in this case is much lower than in the previous model cases. Particularly, the value for saturation occupancy *θ*_∞_ is 0.4576 ± 0.0003, smaller than the value 0.499 ± 0.003 for the Gaussian Hole.

The interpretation is straightforward, given the analysis of the previous sections: the contribution to *B*(*θ*) from the hole-like structures, i.e. valleys, is greater than the contribution from the peaks. Therefore, the occupancy level is lower than obtained in the case of the flat surface.

It is worth mentioning that the resolution of the AFM image introduces an experimental uncertainty that is greater than the theoretical error associated with our modeling approximations. Comparing the image presented in Fig. 10 to the figures reported by D. H. K. Nguyen^9^, it can be seen that the real pillars are steeper, while the AFM data introduces smoothing effects due to the cantilever jumping between the tips of the surface nanopillars. Nevertheless, our approach emphasizes the importance of considering the influence of topology in the process of protein adsorption, and how the geometry defines both the final configuration as well as the pattern of adsorption.

### The timescale of HSA adsorption onto nanostructured surfaces

**Figure 12:**
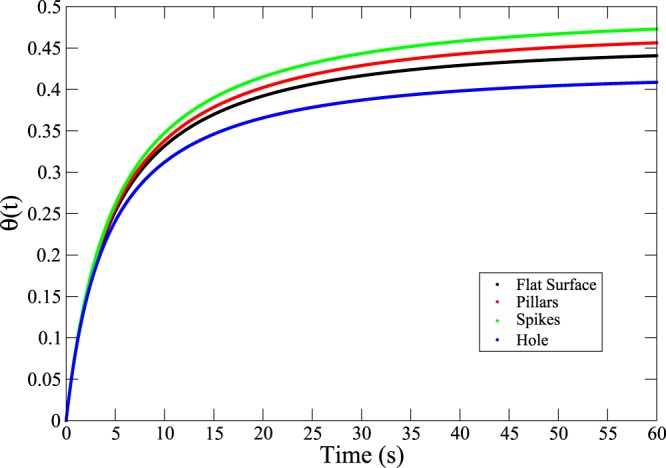
Coverage as a function of time for the four surfaces investigated (*n* = 1.06⋅10^−5^ mol/L).

**Figure 13:**
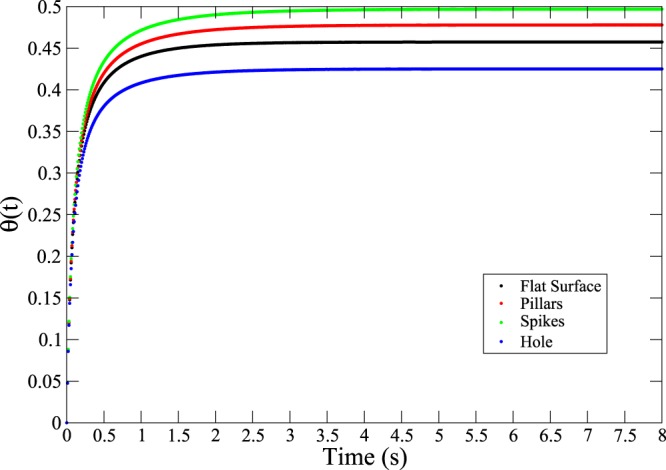
Coverage as a function of time for the four surfaces investigated (*n* = 5.3 ⋅ 10^−4^ mol/L).

An interesting question that arises in practical adsorption experiments concerns the time required for proteins to adsorb onto a substrate surface. The calculations performed in this section aim to provide an insight into the influence of morphology on the adsorption process; therefore specific protein-substrate interactions are neglected. Numerically solving Eq.(1) for albumin taken at the concentration corresponding that in blood plasma (*n* = 5.3 · 10^−4^ mol/L) and an arbitrary lower concentration (*n* = 1.06 · 10^−5^ mol/L) leads to the solutions shown in Figs 12 and 13.

The curves rapidly reach plateau values, which are dependent on the jamming limit. Saturation of the surface is reached in tens of seconds at low protein concentration, and a few seconds at the higher protein concentration corresponding to that found in blood plasma. Since the desorption constant *k*_*d*_ = 5.78 · 10^−4^ s^−1^ is small compared to the adsorption *k*_*a*_ = 10^4^ Lmol^−1^s^−1^^ 17^, the plateau values are very close to those reported in Table 1, which is in line with the assumption that irreversible (or almost irreversible) adsorption is taking place.

Note however, that our model does not take into account the diffusion time *τ*_*D*_ required by the proteins to reach the surface. For the aforementioned approximations to hold, this time scale needs to be smaller than the time scale of adsorption *τ*_*a*_. The diffusion time can be estimated as *τ*_*D*_ = *h*^2^/*D*_dif_, where *D*_dif_ is the diffusion coefficient. For albumin, this assumes the value^20^ *D*_*dif*_ = 2.15 · 10^−11^ m^2^/s, and *h* is the typical length scale of the interaction between proteins and surface. Diffusion depends on the geometry and, thus, the quantity *h* will be, in general, a function of the surface structure. For an estimate value of *h*, however, we can consider a volume *hA* close to the surface, where the density of non-adsorbed proteins is *n*. Then, equating the amount of protein adsorbed to the surface to the amount still in this interaction range, we obtain

6 $$\frac{A}{\sigma }=nhA$$

with *σ* being the cross-section area of albumin. Diffusion is negligible if:

7 $${\tau }_{a}\gg {\tau }_{D}=\frac{1}{{n}^{2}{\sigma }^{2}{D}_{dif}}$$

This yields, for the two concentrations in Figs 12 and 13, ${\tau }_{D}\simeq 0.8\,s$ and ${\tau }_{D}\simeq 3.1\cdot {10}^{-4}\,{\rm{s}}$. Thus, for albumin at the concentration found in blood, the timescale of adsorption is a good approximation of the overall time required to cover the surface with the protein. At lower concentrations, an important contribution to the time of adsorption comes from the time required for the proteins to diffuse to the surface. Therefore, a different approach, coupling the diffusion equation to the RSA model, like the generalized RSA model^20^, would lead to the calculation of more accurate timescales in the case of low protein concentrations. In the case of albumin adsorption at realistic concentration values, however, the simple RSA model allows good estimates of typical timescales to be determined.

### How different would the adsorption results be from those predicted using the Langmuir adsorption model?

**Figure 14:**
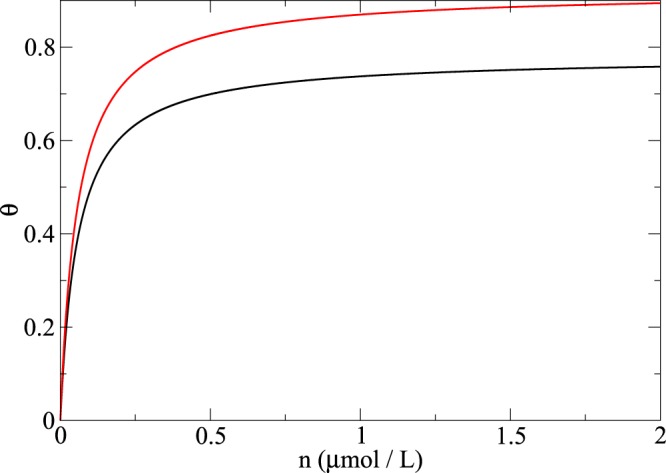
Langmuir adsorption isotherms on flat (black) and nanostructured surface composed of cylinders (red).

Since Langmuir adsorption isotherms are widely used for modelling experimental data, we compared the results obtained by the Langmuir model with those obtained using the RSA model. Langmuir isotherms were obtained from Eq. 2, using the typical value *K*_*eq*_ = 1.73 × 10^7^ L/mol of the adsorption constant, which is valid for albumin^17^. The saturation occupancy *θ*_∞_ was estimated as an upper boundary, assuming hexagonal packing. For a flat surface, this becomes ${\theta }_{\infty }=\frac{\pi }{4}\simeq 0.7854$ and for a nanostructured surface made of hypothetical cylindrical pillars, *θ*_∞_ = 0.92. These are estimated values for the purpose of comparing the Langmuir and RSA models. The resulting isotherms are presented in Fig. 14, which show how the two curves differ, mainly with regard to the coverage saturation limit.

**Figure 15:**
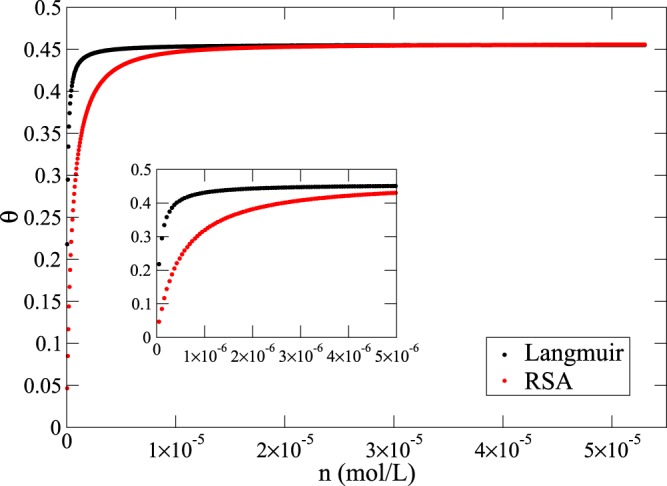
RSA isotherm (red) in comparison with the Langmuir isotherm (black) for spherical proteins adsorbing onto a flat surface.

The different saturation values *θ*_∞_ obtained via the Langmuir adsorption with respect to the jamming limits in Table 1 provides an insight into the departure expected from such an approach in case of irreversible adsorption processes. Even if it is assumed that the Langmuir saturation value matches that of the RSA model, fitting the data using the Langmuir model would be incorrect, as shown in Fig. 15.

The plot shows a steady solution of Eq. (1) for several values of concentration *n*, as is common in the Langmuir approach. While the coefficients *k*_*a*_ and *k*_*d*_ used to derive the two curves are set to the same value in both cases, the Langmuir isotherm reached saturation at a lower concentration than that obtained using the RSA model (*B*(θ) from Table S1). Conversely, if the Langmuir model was used to fit the RSA data, the resulting adsorption and desorption constants would likely lead to wrong interpretations of adsorption behaviour being made^17^.

## Conclusions

In this work we provided a theoretical framework for RSA adsorption on nanostructured surfaces by modeling the adsorption of albumin on periodic arrays of Gaussian pillars, spikes and holes and on a real irregular black silicon surface. Since the results of the RSA simulations are based only on the geometrical features of the surface and on the dimensions of the proteins, the results can be readily generalized for an arbitrary, complex nanostructured surface, and for other globular proteins. Moreover, this rather general analysis can also be applied to topologically similar problems, such as spherical nanoparticle adsorption. The model can be readily generalized to specific adsorption by introducing specific sites instead of homogenous surface or other shapes of proteins.

The results obtained here demonstrated the relationship between the adsorption of globular proteins and the topographical features (height, width and distance between nanostructures) present on substrate surfaces. The increasing saturation occupancy that is associated with increased degrees of substrate curvature can be properly justified by considering how protein *packing* is dependent nanostructure geometry. This modelling provides an accurate estimate of the corresponding blocking function, which was obtained using Monte Carlo simulations taking into account the packing problem of spheres onto a surface. The method described here allowed estimates of the timescales of adsorption to be obtained, which gives, in the case of blood concentrations of albumin, an adsorption rate of the order of 10 seconds for saturation of the surface. This estimate should hold for other nanostructured surfaces.

Our work represents a first step towards the theoretical understanding of the adsorption of proteins onto nanostructured surfaces. Further effort could be devoted to investigating the impact of other relevant properties, such as different protein shapes (e.g. spheroids or polymer filaments), other complex surface geometries and diffusion-adsorption coupling on the process of adsorption.

## Supplementary information

LaTeX Supplementary File
