# Supplementary material for: Simulations of Protein Adsorption on Nanostructured Surfaces
Source: Sci Rep. 2019 Mar 18;9:4694. doi: 10.1038/s41598-019-40920-z (PMC6423022; doi:10.1038/s41598-019-40920-z)
Supplement: Supplementary file 1 — LaTeX Supplementary File [file 41598_2019_40920_MOESM1_ESM.pdf]

Simulations of Protein Adsorption on Nanostructured Surfaces - Supplementary Material

Berardo M. Manzi[1], Marco Werner[2], Elena P. Ivanova[3], Russell J. Crawford[3], and Vladimir A. Baulin[1,*]

[1]Universitat Rovira i Virgili, Departament d'Enginyeria Quimica, Tarragona, 43007, Spain
[2]Leibniz-Institut für Polymerforschung Dresden e.V., Institut Theorie der Polymere, 01069 Dresden, Germany
[3]RMIT University, School of Science, Melbourne, VIC 3001, Australia
[*]vladimir.baulin@urv.cat
[*]va.baulin@gmail.com

Langmuir adsorption on flat surfaces

The Langmuir adsorption model assumes a fixed number of sites that can be occupied reversibly and the blocking function takes a simple form, $B(\theta) = 1 - \theta$. Therefore, Eq.(1) yields

$$\frac{d\theta}{dt} = k_a n(1-\theta) - k_d\theta \tag{S1}$$

After a transient time, adsorbed proteins and proteins in the bulk reach a dynamic equilibrium (stationary state), described by $d\theta/dt = 0$, leading to

$$\theta = \frac{\frac{k_a}{k_d}n}{1+\frac{k_a}{k_d}n} = \frac{K_{eq}n}{1+K_{eq}n}, \tag{S2}$$

with $K_{eq} = k_a/k_d$ being the equilibrium adsorption constant. The finite size of molecules adsorbing on a surface with continuous distribution of adsorption sites can be included in this model by defining θ as the surface fraction occupied by the adsorbed molecules, or

$$\theta = \frac{N_{f_{ad}}\sigma}{A_f}. \tag{S3}$$

Here, $N_{f_{ad}}$ represents the number of particles adsorbed to the surface, A_f is the total area of the adsorbent and $\sigma = \pi r^2 = \pi R_H^2$ is the cross-section area with the radius r equal to the Stokes' radius R_H of a spherical protein.

Such general description provides an universal model of surface adsorption of spherical particles, where all chemical details and molecular interactions are hidden in the equilibrium constant K_{eq}, while the adsorption process is purely kinetic.

Langmuir adsorption on nanostructured surfaces

The model can be extended to the case of nanostructured or convex geometries by taking into account the change of the total available area with respect to a flat surface. Denoting the area of flat surface as A_f and the area of the convex surface as A_s, the maximum number of available sites on flat surface as $N_{f_{tot}}$ and on concave surface as $N_{s_{tot}}$, the size of the site σ, we get the following relations

$$\theta = \frac{N_{\alpha_{ad}}\sigma}{N_{\alpha_{tot}}\sigma}, \tag{S4}$$

$$N_{\alpha_{ad}} = \frac{A_\alpha}{\sigma}\frac{K_{eq}n}{1+K_{eq}n}, \tag{S5}$$

where $\alpha = f$ or s. Thus, for a given protein concentration n, we obtain

$$\frac{N_{s_{ad}}}{N_{f_{ad}}} = \frac{A_s}{A_f}. \tag{S6}$$

These relations represent an oversimplified picture of the adsorption process for several reasons, one of which is purely geometrical in nature. Consider, for the sake of simplicity, an unidimensional adsorption-like process of circles on a line of length L, Fig. 1.

The maximum amount of circles which can be placed on the line is $N_{f_{max}} = L/2r$. Now, if the circles adsorb on a spherical shell of same length $L = 2\pi R$, the maximum amount of particles will be defined as $N_{s_{max}} = L/s$, where s is the arc length:

$$s = \alpha R = 2 \arcsin\left(\frac{r}{r+R}\right) R, \tag{S7}$$

which is smaller than $2r$, $s < 2r$, provided that:

$$\frac{r}{r+R} < \sin\left(\frac{r}{R}\right) < \sin(1) \approx 0.84, \tag{S8}$$

or

$$r < 5.25R, \tag{S9}$$

which certainly holds if $R > r$. Therefore, a convex shape will increase the amount of total occupied area resulting in a higher *packing* per surface area. This trend has been confirmed in experiments of adsorption of albumin on titanium surfaces[Scopelliti et al.(2010)].

To take into account the *packing* of spheres on a generalized surface, Eq. (S1) can be rewritten as:

$$\frac{d\theta}{dt} = k_a n(\theta_\infty - \theta) - k_d \theta, \tag{S10}$$

where θ_∞ is the maximum coverage allowed by packing and $B(\theta) = \theta_\infty - \theta$. This leads to a modified version of Eq. (S2):

$$\theta = \frac{K_{eq} n}{1 + K_{eq} n} \theta_\infty. \tag{S11}$$

The applicability of the Langmuir approach is nevertheless very limited, mainly due to the assumption of reversible adsorption. Indeed, very often the protein adsorption is *irreversible* or almost irreversible[Vogler(2012)], and the fitting of the experimentally obtained isotherm to a Langmuir isotherm results in unrealistic adsorption constants[Latour(2015)].

Description of RSA simulations

Analytical solutions for Random Sequential Adsorption are very rare, and only a few specific problems can be tackled in this way. Generally, Monte Carlo (MC) simulations are employed to derive numerically properties such as the Blocking function $B(\theta)$ and the jamming limit θ_∞.

The simulations are implemented in an in-house deployed software. Each simulation consists in sequential attempts to place a single, spherical particle onto surface, with the attempt deemed as failed if another particle is already placed at distance $d < 2r$. The number of attempts N_{att} required to attack particle i is recorded, while the number of successful attachments, per single simulation, is always 1. Each of the seven configurations described in the main text is repeated 1000 times, and the average number of attempts for the i-th particle is computed, yielding Eq. (13), reported here for convenience:

$$B(\theta) = \frac{N_{succ}}{N_{att}}.$$

The jamming limit θ_∞ is computed as the average of the final occupancy for each of the simulations. Notice that, due to the random nature of the simulations, the final amount of particles adhering to the surface varies between any two simulations. Moreover, an upper bound to the possible amount of attempts per particle is defined, such that no simulation will indefinitely run when the surface is completely covered.

Concerning the placement of a single particle, a few details are relevant. First, being the surface defined on a rectangular support, i.e. $x_{lo} \leq x \leq x_{hi}$ and $y_{lo} \leq y \leq y_{hi}$, where x_{lo} and y_{lo} are the lower bounds for the x and y coordinates, respectively, and x_{hi}, y_{hi} are the upper, a particle placed on said border is considered periodically wrapped to the respective opposite edge. Such periodic boundary conditions allow to simulate a single pillar or spike, while considering it part of a periodic array.

Second and more importantly, the random generation of particles is required to be uniformly distributed on the surface. This is of no concern on flat surfaces, where uniform generation of random number obtained by independent generation of x and y coordinates. However, when considering curved surfaces, the same approach would fail, as uniform generation of random

Table S1: Parameters used to fit $B(\theta)$ of all the simulations set illustrated in this work. The fit is defined as $B(\theta) = a_0 + a_1\theta + a_2\theta^2 + a_3\theta^3$.

	H (nm)	W (nm)	a_0	a_1	a_2	a_3
Flat	-	-	1.0063	−4.2007	4.5347	−0.3509
Pillars	150	80	1.0067	−4.1564	4.5746	−0.6089
	200	60	1.0095	−4.0085	4.3987	−0.5408
	200	70	1.0053	−4.0674	4.3203	−0.4412
	200	80	1.0079	−4.1356	4.5255	−0.5957
	200	90	1.0080	−4.1779	4.6793	−0.7364
	200	100	1.0076	−4.2060	4.7735	−0.8094
	250	80	1.0076	−4.1053	4.4484	−0.5591
Spikes	450	60	1.0076	−3.9650	4.0982	−0.4055
	500	40	1.0061	−3.8784	3.9725	−0.4624
	500	50	1.0076	−3.9109	3.9752	−0.3649
	500	60	1.0061	−3.9430	4.0555	−0.3922
	500	70	1.0069	−3.9881	4.1535	−0.4192
	500	80	1.0069	−4.0318	4.2795	−0.5000
	550	60	1.0068	−3.9227	3.9703	−0.3088
Hole	-550	60	1.0049	−4.5960	5.5021	−0.5908

numbers on the xy plane would not imply uniform generation on the surface. Indeed, the probability dP to attach is proportional to the *metric density* g of the surface, or in other words:

$$dP \propto \sqrt{g}dxdy.$$

For a flat surface $g = 1$ and the distribution is uniform, while for the gaussian shape of Eq.(14):

$$g = 1 + \frac{H^2}{v^2} \exp\left(-\frac{(x-x_i)^2 + (y-y_i)^2}{v}\right).$$

Evidently, this same relation defines the distribution which can be used for generating random numbers; it is sufficient to invert it to obtain a stochastic variable from the distribution. Unfortunately, the inversion of this relation is hard (if not impossible) and a different approach has been exploited in our work.

The x and y coordinates of the position onto which to attempt to attach the next particle is generated uniformly, as in the flat plane case. Then, a uniform random number $0 \le R \le 1$ is generated, and the position is taken into account if $R < g(x,y)$. Notice that if this condition is not met, it does not count as a placement attempt, and N_{att} is not increased. This method allows for a uniform position generation on the underlying surface, at the expanse of a slightly higher computational cost.

Fitting parameters for the blocking function

Table S1 lists the fitting parameters for the blocking function for the different configurations mentioned in the main text.

References

Scopelliti et al.(2010). Scopelliti, P. E.; Borgonovo, A.; Indrieri, M.; Giorgetti, L.; Bongiorno, G.; Carbone, R.; Podestà, A.; Milani, P. The effect of surface nanometre-scale morphology on protein adsorption. *PLoS One* **2010**, *5*, 1–9.

Vogler(2012). Vogler, E. A. Protein adsorption in three dimensions. *Biomaterials* **2012**, *33*, 1201–1237.

Latour(2015). Latour, R. A. The Langmuir isotherm: A commonly applied but misleading approach for the analysis of protein adsorption behavior. *J. Biomed. Mater. Res. - Part A* **2015**, *103*, 949–958.
